# Functional variants regulating *LGALS1 (Galectin 1)* expression affect human susceptibility to influenza A(H7N9)

**DOI:** 10.1038/srep08517

**Published:** 2015-02-17

**Authors:** Yu Chen, Jie Zhou, Zhongshan Cheng, Shigui Yang, Hin Chu, Yanhui Fan, Cun Li, Bosco Ho-Yin Wong, Shufa Zheng, Yixin Zhu, Fei Yu, Yiyin Wang, Xiaoli Liu, Hainv Gao, Liang Yu, Linglin Tang, Dawei Cui, Ke Hao, Yohan Bossé, Ma′en Obeidat, Corry-Anke Brandsma, You-Qiang Song, Kelvin Kai-Wang To, Pak Chung Sham, Kwok-Yung Yuen, Lanjuan Li

**Affiliations:** 1 State Key Laboratory for Diagnosis and Treatment of Infectious Diseases, First Affiliated Hospital, College of Medicine, Zhejiang University; 2 Collaborative Innovation Center for Diagnosis and Treatment of Infectious Diseases, Hangzhou, China; 3 State Key Laboratory of Emerging Infectious Diseases; 4 Department of Microbiology; 5 Research Centre of Infection and Immunology; 6 Carol Yu Centre for Infection; 7 Department of Biochemistry, The University of Hong Kong; 8 Department of Genetics and Genomic Sciences, Icahn School of Medicine at Mount Sinai, USA; 9 Department of Molecular Medicine, Laval University, Canada; 10 The University of British Columbia Center for Heart Lung Innovation, St Paul's Hospital, Canada; 11 University of Groningen, University Medical Center Groningen, Department of Pathology and Medical Biology, Groningen Research Institute for Asthma and COPD, The Netherlands; 12 Centre for Genomic Sciences, The University of Hong Kong, Hong Kong

## Abstract

The fatality of avian influenza A(H7N9) infection in humans was over 30%. To identify human genetic susceptibility to A(H7N9) infection, we performed a genome-wide association study (GWAS) involving 102 A(H7N9) patients and 106 heavily-exposed healthy poultry workers, a sample size critically restricted by the small number of human A(H7N9) cases. To tackle the stringent significance cutoff of GWAS, we utilized an artificial imputation program SnipSnip to improve the association signals. In single-SNP analysis, one of the top SNPs was rs13057866 of *LGALS1*. The artificial imputation (AI) identified three non-genotyped causal variants, which can be represented by three anchor/partner SNP pairs rs13057866/rs9622682 (AI *P* = 1.81 × 10^−7^), rs4820294/rs2899292 (2.13 × 10^−7^) and rs62236673/rs2899292 (4.25 × 10^−7^) respectively. Haplotype analysis of rs4820294 and rs2899292 could simulate the signal of a causal variant. The rs4820294/rs2899292 haplotype GG, in association with protection from A(H7N9) infection (OR = 0.26, *P* = 5.92 × 10^−7^) correlated to significantly higher levels of *LGALS1* mRNA (*P* = 0.050) and protein expression (*P* = 0.025) in lymphoblast cell lines. Additionally, rs4820294 was mapped as an eQTL in human primary monocytes and lung tissues. In conclusion, functional variants of *LGALS1* causing the expression variations are contributable to the differential susceptibility to influenza A(H7N9).

A novel reassortant avian influenza A virus H7N9 [A(H7N9)] has caused human infections since the spring of 2013^1^,^2^,^3^. As of July 7^th^ 2014, there had been a total of 451 laboratory-confirmed cases, including at least 156 deaths^4^, which was about ten-fold of the case number of human H5N1 infection in the past 11 years in China^5^. The over 30% case-fatality rate of A(H7N9) infection is greater than that of the SARS coronavirus outbreak in 2003 and similar to the ongoing MERS coronavirus outbreak^6^,^7^. Clinically, most A(H7N9) patients exhibited lower respiratory tract infection, some of them complicated with multi-organ dysfunction; whilst a small portion of patients, basically children and young adults exhibited mild symptoms^2^,^8^,^9^.

Most A(H7N9) patients had a recent history of poultry contact^5^. In fact, exposure to domestic poultries is not rare in both rural and urban settings^10^. Many studies demonstrated that the pathogenicity of influenza virus is codetermined by both host and virus genetics. Since the A(H7N9) outbreak, multiple isolates have been recovered from human, poultry and environment, designated into more than twenty genotypes. Moreover, the genetic heterogeneity increased along with virus spread and transmission^11^. However, there has been little evidence showing the remarkable difference in virulence or infectivity among various strains. Therefore, we speculated whether human genetic variations may cause differential susceptibility to A(H7N9) infection. Human genetic predisposition to influenza infection and development of severe disease upon infection has been underscored by World Health Organization and the scientific community. The familial clustering of human A(H5N1) infection among blood relatives argued for a possible genetic susceptibility to the infection^12^. Furthermore, accumulating evidence demonstrated that human genetic polymorphisms contribute to the outcome and disease severity of influenza infection. We and others have demonstrated that genetic polymorphisms of *IFITM3, SFTPB* and *CD55* gene may contribute to the disease severity of 2009 pandemic H1N1 influenza^13^,^14^,^15^. The same variant, rs12252 of *IFITM3*, was associated with disease progression of human H7N9 infection^16^. However, the comprehensive understanding of genetic susceptibility to novel A(H7N9) infection remained largely elusive.

Genome-wide association study (GWAS) has been widely utilized to uncover the genetic basis of human diseases. However, compared to other prevalent diseases, the relative small number of human A(H7N9) influenza cases posed a considerable difficulty to the study of genetic susceptibility to the infection. With a total of less than 400 cases of human A(H7N9) infection when we started the study, we managed to obtain the genomic DNA samples of 102 Chinese patients from Southern China. With a sample size at this level, it is unlikely to identify an association variant with genome-wide significance, i.e. 5 × 10^−8^, unless the effect size is very large (e.g. odds ratio of more than 3). Despite the existing difficulty, we proceeded to GWAS since it allows the comprehensive understanding of the possible genetic determinism of human A(H7N9) influenza in an unbiased manner. We performed a GWAS in 102 A(H7N9) patients and 106 local healthy poultry workers, who have been intensively exposed to A(H7N9) viruses without clinical evidence of infection. To tackle the stringent significance cutoff of GWAS, we utilized various approaches to analyze GWAS data. We applied an artificial imputation program SnipSnip^17^ to improve the association signals and facilitate the discovery of association variants. We performed pathway-based analysis to uncover the biological pathways associated with the higher risk to A(H7N9) infection. The expression quantitative trait loci (eQTL) mapping was also leveraged to facilitate the identification of the association variants. We demonstrated that genetic variations in lectin, galactoside-binding, soluble, 1 (*LGALS1*, also known as *Galectin 1*) are contributable to the differential susceptibility to A(H7N9) influenza. Moreover, two biological pathways, extracellular matrix (ECM)-receptor interaction and mitogen-activated protein kinase (MAPK) signaling are significantly enriched pathways associated with the susceptibility to influenza A(H7N9) infection.

## Results

### Epidemiological characteristics of A(H7N9) patients and poultry workers

A total of 102 laboratory-confirmed A(H7N9) patients were included in this study (Table 1). Among these patients, 98% (100/102) required oxygen supplementation, 92% (94/102) were admitted to the intensive care unit, 73% (74/102) had acute respiratory distress syndrome (ARDS), 32% (33/102) had multi-organ dysfunction syndrome (MODS) and 26% (27/102) died. When compared to those who survived, patients who died were significantly older, with more incidence of chronic pulmonary diseases and significantly higher levels of urea, creatinine, bilirubin, aspartate transaminase, lactate dehydrogenase, creatine kinase, C-reactive protein and d-dimer, as well as the higher Acute Physiology and Chronic Health Evaluation (APACHE) II score, but significantly lower levels of hemoglobin.

A total of 106 healthy poultry workers were included as controls. These healthy poultry workers were intentionally recruited since they were the most comparable population to A(H7N9) patients in terms of the virus exposure, the key determinant for human A(H7N9) infection. There was no significant difference in the proportion of female between the A(H7N9) cases and poultry worker controls (34% (35/102) vs 35% (37/106), *P* = 1.000). However, the A(H7N9) patients were significantly older than the poultry worker controls (median age: 61 years [interquartile range, 50 ~ 68 years] vs 48.5 years [interquartile range, 46 ~ 53 years]; *P* < 0.001). Our attempt to recruit age-comparable controls for the patients was unsuccessful due to the job requirement for poultry workers; since poultry trafficking and transaction involve numerous physically-challenging labors, which would be too demanding for people at their sixties.

### Identification of genetic variants associated with A(H7N9) infection

**Figure 1:**
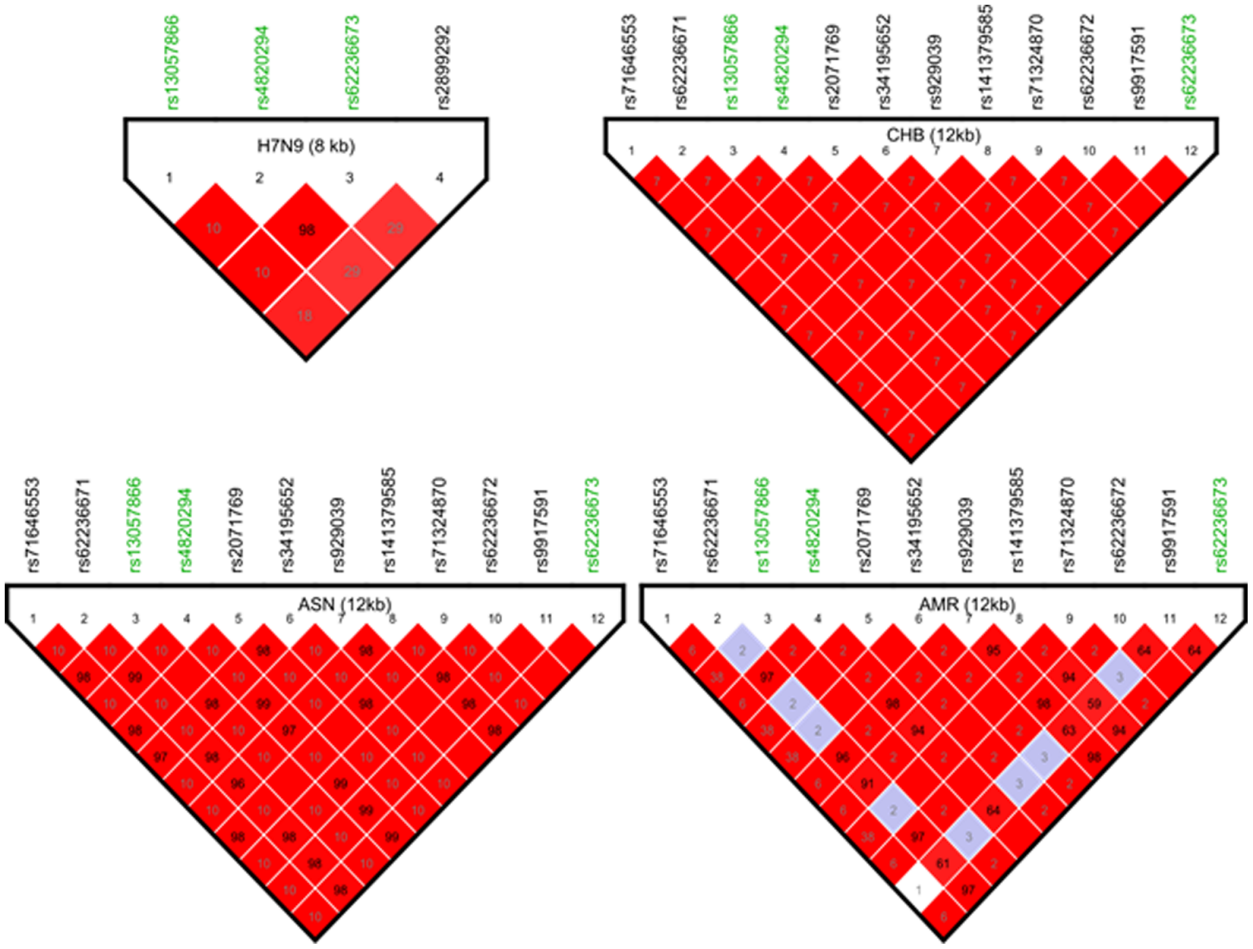
Linkage disequilibrium pattern of three anchor SNPs rs4820294, rs13057866, rs62236773 and the related variants. The LD pattern of three anchor SNPs rs13057866, rs4820294, rs62236673 (in green color) and partner SNP rs2899292 are plotted for 208 study participants in this study (H7N9). The LD patterns of the three anchor SNPs and their high LD variants are plotted for 94 individuals from Chinese Han in Beijing (CHB), 264 individuals from Asian (ASN), and 172 individuals from America (AMR), whose genotypes are retrieved from 1000 Genomes Project. The boxes are colored according to D′ measure on a white and red scale where red indicates complete LD (D' = 1). The numbers inside the boxes are r^2^ measure. The red box without number indicates the highest r^2^ of 1.0.

HumanOmniZhongHua-8 BeadChips were applied to genotype all study participants, with genotype calling rate >99%. The genome-wide genotyping data were analyzed for the allelic association. The top SNP, rs1960384, an intronic variant in *C8B* gene, and rs13057866, a SNP 2 kb upstream of *LGALS1*, had allelic association *P* value of 2.07 × 10^−6^ and 2.75 × 10^−6^, respectively. As expected, none of SNPs met the significance threshold for GWAS. We performed the standard imputation in the study participants, in 0.2 Mb region around the two candidate loci in *C8B* and *LGALS1*. The top SNPs were rs1960384 (*C8B*) and rs71646553 (supplementary Table 1). rs71646553, an indel 8 kb upstream of *LGALS1,* surfaced from the imputation with slightly increased association signal (*P* = 2.72 × 10^−6^), probably due to its high linkage disequilibrium (LD) with rs13057866 (Figure 1, r^2^ = 1.0 in Chinese and r^2^ = 0.98 in Asian).

**Figure 2:**
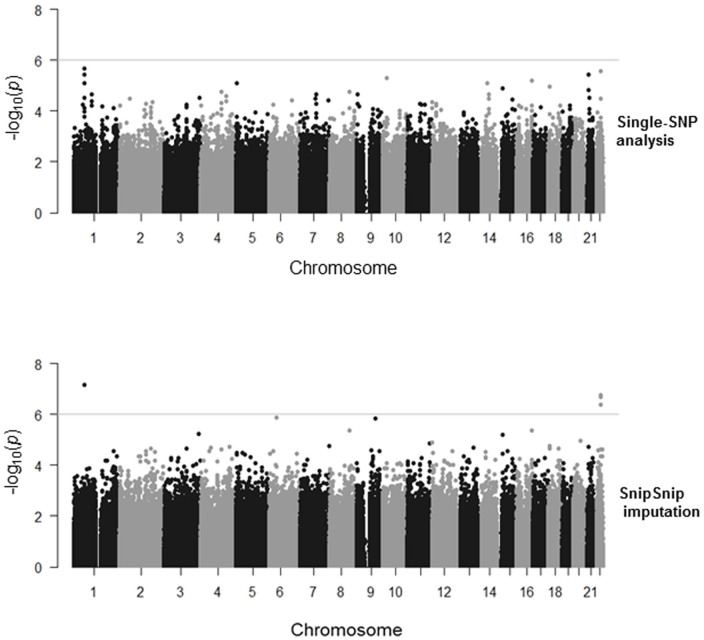
The artificial imputation implemented in SnipSnip increased the association signal compared with single-SNP analysis. Manhattan plots show *P* values of SNPs (y-axis, -log_10_ scale) on a genomic scale (x axis) of A(H7N9) GWAS dataset. The single-SNP allelic association *P* values using logistic regression implemented in PLINK and artificial imputation *P* values using SnipSnip are shown in the upper and lower panel respectively.

A software implementation, SnipSnip, has been recently designed to increase the association signal of poorly-tagged or non-genotyped causal variant by combinational analysis of two genotyped SNPs (an anchor SNP and a partner SNP) in weak linkage disequilibrium with the causal variant^17^. The software uses a sophisticated algorithm to select an optimal nearby partner SNP for each anchor SNP based on the correlation between the two SNPs. In simulation studies, the program successfully identified causal variants which would otherwise be missed by the conventional single-SNP analysis^17^. In this study, all variants in our GWAS dataset were subject to SnipSnip analysis. As shown in Figure 2, SnipSnip implementation substantially increased the association signals compared to the traditional single-SNP analysis. Interestingly, the top 4 causal variants were mapped to *C8B* and *LGALS1* (Table 2), the identical genes discovered in single-SNP analysis as top candidates. The artificial imputation (AI) *P* value (6.72 × 10^−8^) of the top association signal, a variant of *C8B*, marginally met the genome-wide significance threshold; while the AI *P* values of the next three variants, which were mapped to *LGALS1*, fell at 10^−7^ level. Despite the failure to achieve genome-wide significance for variants of *LGALS1*, our results did yield variants with higher statistical significance than would be expected by chance as shown in Q-Q plot of our data (supplementary Figure 1). The two *C8B* variants, rs1960384 (the top SNP) and rs646606 (the anchor SNP) are both intronic SNPs. There has been little evidence implicating the functional alteration related to these two variants. On contrary, the anti-influenza activity of *LGALS1* has been demonstrated in a recent study^18^. In this study, three out of the top four association variants from SnipSnip analysis were mapped to *LGALS1*. Therefore, we focused on *LGALS1* to explore the possible functional mechanism underlying the genetic associations.

### The in-depth analysis of association variants in *LGALS1* and the functional validation

Notably, three variants of *LGALS1* were associated with the susceptibility to A(H7N9) infection (Table 2). Among the three anchor SNPs, rs4820294 and rs62236673 were in high LD in our study participants (r^2^ = 0.98) and other populations (r^2^ = 1.0, Figure 1), indicating the anchor/partner pair rs4820294/rs2899292 and rs62236673/rs2899292 captured the identical causal variant. We chose one pair rs4820294/rs2899292 for further analysis. The causal variant captured by SnipSnip analysis was represented as an anchor/partner pair, which are in weak LD with causal variant but can optimally capture the association signal for the latter. Although AI *P* values of causal variants are illustrated in Table 2, we are unaware of their distribution in the patient and control group; neither can we postulate the possible molecular mechanism underlying the associations. Now that the anchor and partner SNP are in weak LD with the causal variant, we inferred the distribution of the causal variant by haplotype analysis of the anchor and partner SNP although the association and distribution of anchor/partner haplotypes as surrogate for those of non-genotyped causal variant may be an underestimation^17^. We simulated the association signal of the causal variant by haplotype analysis of anchor/partner pair rs4820294/rs2899292. As shown in Table 3, due to the LD between rs4820294 and rs2899292 (Figure 1; A(H7N9)), the haplotype AA was rarely present in our study subjects with an overall frequency of less than 2%. The strongest association signal was haplotype GG, which was significantly overrepresented in controls (35.58%) than in A(H7N9) patients (14.33%). The odds ratio of 0.26 for haplotype GG with the liability to infection can be translated into a more comprehensible interpretation that carriers of this haplotype were conferred 3.84 (95% CI, 2.24–6.58) fold of protection from A(H7N9) infection compared with non-carriers (*P* = 5.92 × 10^−7^). The approximation of the *P* value of rs4820294/rs2899292 haplotype GG to the AI *P* value of rs4820294/rs2899292 (2.13 × 10^−7^) suggested that the haplotype GG can appropriately simulate the signal of causal variant represented by SNP pair rs4820294/rs2899292. The identification of rs4820294/rs2899292 haplotype GG was literally an advantage of SnipSnip over other commonly-used imputation softwares since SnipSnip can capture the association signals when they derive from a genuine haplotype effect but not to the effect of any ungenotyped single-locus variant.

**Figure 3:**
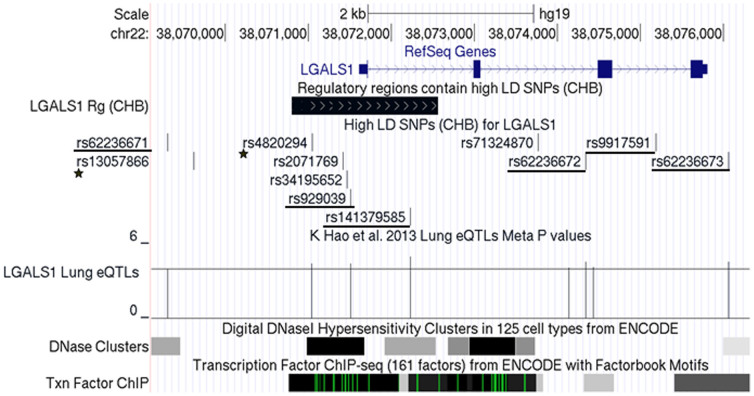
The genetic architecture of *LGALS1* gene. The upper panel denotes the chromosomal region that accommodates the *LGALS1* gene. The region flanking the transcriptional start site is a conserved regulatory region containing high LD SNPs in Chinese and other populations. The variants in high LD with anchor SNPs, rs4820294 and rs13057866 (denoted with ★), are shown in the next panel. The underlined variants are those in high LD with rs4820294 while un-marked ones are in high LD with rs13057866. Lung eQTL panel shows the locations of eQTL and *P* values in -log_10_ scale. The horizontal bar represents the -log_10_*P* value of 4. DNAse I hypersensitivity cluster and transcriptional factor binding site signals are annotated according to the experimental data from ENCODE Consortium. The gray box indicates the extent of the hypersensitive region or cluster of transcriptional factor occupancy. The darkness is proportional to the maximum signal strength observed in any cell line. The green line indicates the highest scoring site of an identified canonical motif for the corresponding factor.

**Figure 4:**
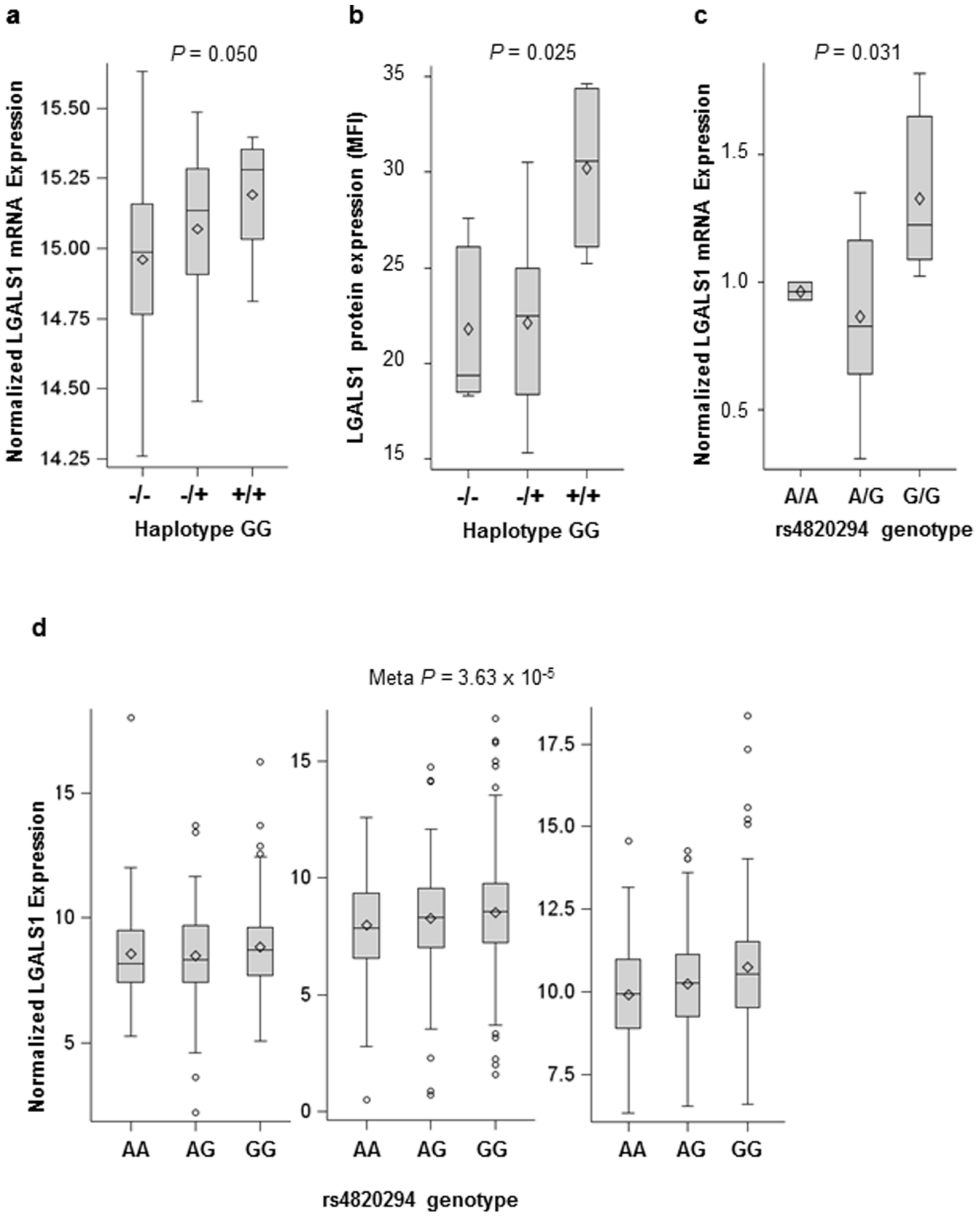
The association variants of *LGALS1* are correlated to differential expression levels in lymphoblastoid cell lines (LCLs), human monocytes and lung tissues. (4a) Boxplot of *LGALS1* mRNA expression according to rs4820294/rs2899292 haplotype GG in LCLs. The carriage of haplotype GG significantly correlated to *LGALS1* mRNA expression (*P* = 0.050) in LCLs generated from 74 Chinese Han from Beijing (CHB). In x-axis, -/-, -/+, and +/+ denote non-carriers (N = 49), heterozygotes (N = 21), and homozygotes (N = 4) of rs4820294/rs2899292 haplotype GG respectively. The box denotes the interquartile range. The line and diamond within the box represent the median and average respectively. Linear regression analysis was used to analyze the data. (4b)Boxplot of *LGALS1* protein expression corresponding to rs4820294/rs2899292 haplotype GG in 21 LCLs (N = 8 for -/-, 9 for -/+ and 4 for +/+) by flow cytometry analysis. Carriage of rs4820294/rs2899292 haplotype GG significantly correlated to the LGALS1 protein expression in these LCLs (*P* = 0.025). MFI, mean florescence intensity. (4c)The anchor SNP rs4820294, a variant in the proximal promoter of *LGALS1*, regulated *LGALS1* mRNA expression. A total of 19 mRNA samples (N = 2 for genotype A/A, 8 for A/G and 9 for G/G) from peripheral blood monocytes expressed differential levels of *LGALS1* transcript in a genotype-specific manner (*P* = 0.031) by RT-qPCR assay. Levels of *LGALS1* transcript are normalized with those of *GAPDH*. (4d) Gene expression levels of *LGALS1* in human lung correlated to genotype groups of rs4820294 (meta-analysis *P* = 3.63 × 10^−5^). The lung cis-eQTL dataset has been generated from lung specimens collected at three centers examining a total of 1111 individuals. Denotations of boxplot are the same as 4a, except that open dots represent the outliers.

In UCSC genome browser (http://genome.ucsc.edu/), the promoter region of *LGALS1* that accommodates rs4820294, is a conserved regulatory region with strong signals for transcriptional factor binding and DNase hypersensitivity based on the annotation of Encyclopedia of DNA elements (ENCODE) Consortium (Figure 3), an international collaboration to functionally annotate human genome. We inferred that rs4820294 and its related variants might affect the expression regulation of *LGALS1*. We retrieved *LGALS1* mRNA expression data of lymphoblast cell lines (LCLs) from 74 Chinese individuals from Genevar (GENe Expression VARiation) eQTL (expression quantitative trait loci) database^19^, and corresponding genotyping data from 1000 Genomes Project, to assess the possible haplotype-expression correlation. We found that rs4820294/rs2899292 haplotype GG exhibited higher *LGALS1* mRNA expression in these 74 LCLs. The *LGALS1* mRNA level in LCLs significantly correlated to the carriage status of haplotype GG, i.e., homozygous-, heterozygous- and non-carriage, by linear regression analysis (*P* = 0.050, Figure 4a & Table 3). To verify the haplotype-expression correlation in the protein level, we chose 21 LCLs with three distinct carriage statuses of rs4820294/rs2899292 haplotype GG and examined intracellular LGALS1 protein expression levels by flow cytometry analysis. Consistent with the result of eQTL mapping, LCLs carrying homozygous haplotype GG exhibited the highest levels of LGALS1 protein (Figure 4b, *P* = 0.025). Therefore, the higher *LGALS1* expression level of rs4820294/rs2899292 haplotype GG may represent the molecular underpinning for its genetic association with the resistance to A(H7N9) infection.

LCLs are immortalized cell lines derived from EBV-transformed human B lymphocytes. We proceeded to examine the *LGALS1* expression in primary human cells and tissues. Based on our observation with flow cytometry, LGALS1 protein levels were higher in peripheral blood monocytes than in lymphocytes (unpublished data). We isolated monocytes from peripheral blood mononuclear cells to assay levels of *LGALS1* transcript by RT-qPCR. We found that rs4820294 *per se* was an eQTL in human peripheral blood monocytes. As shown in Figure 4c, the rs4820294 G/G genotype exhibited the highest transcript level of *LGALS1* in human monocytes (*P* = 0.031). Furthermore, we extracted the cis expression quantitative trait loci (cis-eQTL) for *LGALS1* from the lung eQTL dataset^20^. We demonstrated that rs4820294 was a strong cis-eQTL for *LGALS1* in lung tissues of 1111 individuals (Figure 4d, meta-analysis *P* = 3.63 × 10^−5^). Notably, in the absence of the partner SNP rs2899292, the rs4820294 protective genotype G/G generated an association signal at a *P* value of 5.20 × 10^−4^ with an odds ratio of 2.67 for protection.

In both single-SNP analysis and SnipSnip analysis, rs13057866, a SNP 2 kb upstream of *LGALS1*, emerged as one of the top hits. rs13057866 was in high LD with rs2071769 (r^2^ = 1) and rs34195652 (r^2^ ≥ 0.98) in Han Chinese (CHB), Asian (ASN) and American (AMR, Figure 1). rs34195652, captured by our in-house generated program IndelLDplot, is a T/TG indel 154 bp upstream *LGALS1*. As shown in Figure 3, the region harboring rs2071769 and rs34195652 has been defined as a strong regulatory region according to the experimental evidence of ENCODE Consortium. According to the functional annotation of HaploReg 2, the indel alters the regulatory motif of many putative transcription factors. It has been demonstrated that the eQTLs proximate to the transcription start size tend to be more conserved cross cell types and have stronger effect^19^. Therefore, rs2071769 and rs34195652 are very likely the functional variants that can tag rs13057866.

As mentioned above, in the single-SNP analysis, rs13057866 displayed a strong association signal with an allelic association *P* value of 2.75 × 10^−6^. The protective allele A was over-represented in control group (29.3%) than in patient group (10.8%) with an odds ratio of 0.29, which can be translated into 3.41 fold higher protection from A(H7N9) infection to carriers of the allele A than the non-carriers. Actually, rs13057866 A allele can mark rs4820294/rs2899292 GG haplotype due to the complete linkage between rs13057866 and rs4820294 (D' = 1.0) in all the queried populations (our cohort, ASN, AMR and CHB) as well as the high LD between rs13057866 and rs2899292 in our study cohort (D' = 0.92) and other populations (D' ≥ 0.74, Figure 1). Specifically, rs13057866, rs4820294 and their high LD variants form a high LD block in the regulatory region of *LGALS1* (Figure 1). Based on experimental evidence of ENCODE, most of these variants are functional (supplementary Table 2). Namely, the variants aggregated in the regulatory region form a functional haplotype which may affect *LGALS1* gene expression. As mentioned above, the standard imputation in the region accommodating *LGALS1* failed to obtain any variant with dramatically increased association signal than rs13057866. Therefore, the “non-genotyped causal variant” captured by SnipSnip implementation is unlikely a single-locus variant. Instead, the functionally haplotype(s) tagged by rs13057866, rs4820294 and their high LD variants are the real causal variants which generated the strong association signals in SnipSnip implementation (Table 2). Collectively, integrating our experimental findings and data mining from the public databases, we demonstrated that rs4820294, rs13057866 and their high LD variants may jointly encode *LGALS1* expression variations, thereby caused the differential susceptibility to A(H7N9) infection among humans.

### The identification of biological pathways associated with A(H7N9) infection

Pathway-based analysis or gene set enrichment analysis have emerged as a powerful approach to overcome the limitation of traditional single-SNP analysis of GWAS^21^, based on the concept that genes do not work in isolation; functionally related genes in the same molecular network or pathway are often involved in the disease susceptibility^22^. In contrast with single-SNP association, pathway-based analysis examines groups of functionally related genes, each of which may have too small effect to be detected individually, but can be detectable when analyzed as a functional group. In our study, all the variants with *P* < 0.001 were mapped to 536 unique genes with ANNOVAR^23^, which were then applied to a WEB-based GEne SeT AnaLysis Toolkit (WebGestalt)^24^. WebGestalt was used to identify the enriched pathways in association with the susceptibility to A(H7N9) infection in Kyoto Encyclopedia of the Genes and Genomes (KEGG) pathway collection, which consists of 390 well-defined categories. The top two hits were the extracellular matrix (ECM)-receptor interaction and mitogen-activated protein kinase (MAPK) signaling pathway (Table 4). For example, there are totally 85 reference genes in the ECM-receptor category. Among our gene set of 536 genes, 8 polymorphic genes fell in this category while the expected number is 1.03 by chance. After correction for the multiple tests, the ECM-receptor network represents a significantly enriched category with an enrichment ratio of 7.76 and an adjusted *P* value of 0.0008. Similarly, 12 genes in our gene set were components of MAPK signaling pathway with the enrichment ratio of 3.69 (adjusted *P* value = 0.0045). Therefore, the gene set analysis suggested that ECM-receptor interaction and MAPK signaling were the significantly enriched pathways associated with the increased susceptibility to the A(H7N9) infection.

## Discussion

In this GWAS, we identified two polymorphic genes *LGALS1* and *C8B* as well as two biological pathways, ECM-receptor interaction and MAPK signaling pathway, which are significantly associated with the susceptibility to A(H7N9) infection. GWAS has been successfully performed to identify the genetic variations associated with the development of human diseases in an unbiased approach^25^. However, the stringent genome-wide significance threshold and the modest genetic effect size may mask the real associations^26^. Both experimental and computational data support the notion that a considerable proportion of trait-associated loci harbor variations that impact the abundance of specific transcripts^27^. These expression-related variations are referred as expression quantitative trait loci (eQTLs), which have been mapped by paralleled genome-wide analysis of gene expression and genetic variations in LCLs^28^ and various human tissues^20^,^29^. The local eQTLs (cis-eQTLs) refer to those within 1 Mb to the regulated gene. cis-eQTLs tend to have larger effect on gene expression than distant eQTLs (trans-eQTLs)^29^. The availability of systematically generated eQTLs has been widely leveraged to prioritize the discovery of GWAS, facilitate the identification of causal genes and provide insight into the biological basis for the identified disease associations^20^,^30^. In this study, the identification of rs4820294/rs2899292 haplotype GG and rs4820294 as eQTLs for *LGALS1* in LCLs and primary human cells adequately substantiates their genetic association with the susceptibility to A(H7N9) infection. These results invariably underscore the role of *LGALS1* to protect from human A(H7N9) infection.

LGALS1 contains conserved carbohydrate recognition domains to distinctive patterns of carbohydrates on the surfaces of various microorganisms^31^. A recent study demonstrated that LGALS1 can bind to various subtypes of influenza A viruses, inhibiting viral infectivity and viral production. The mouse experiments showed that treatment with LGALS1 reduced viral load, attenuated lung inflammation and increased mouse survival. Moreover, the *LGALS1*-knockout mice were more susceptible to the fatal influenza infection than wild-type mice^18^. According to a public database Genevestigator, human *LGALS1* is highly expressed in plasma, variety of cells and tissues, including upper and lower respiratory epithelium. The highly expressed *LGALS1* in bronchial epithelial cells displayed considerable variations among 215 humans. In this study, we demonstrated that genetic variants of *LGALS1*, including rs4820294 and rs13057866 and the related variants, encode the higher expression of *LGALS1* in humans, which may confer the carriers of these variants more protection from A(H7N9) infection.

Our analysis revealed that two biological pathways, ECM-receptor interaction and MAPK signaling, were significantly enriched pathways associated with the susceptibility to human A(H7N9) infection. The extracellular matrix consists of a complex mixture of functional macromolecules and provides structural and functional support to the surrounding cells, including proteoglycans, non-proteoglycan polysaccharide, collagens, fibronectin and laminin etc. The ECM interacts with transmembrane receptors to mediate cellular activities such as adhesion, migration, differentiation, proliferation and apoptosis. For example, fibronectins bind collagen and cell-surface integrins, re-organize cell cytoskeleton to facilitate cell movement. ECM and interacting proteins have been implicated in the entry of various viruses including gamma-retrovirus, hepatitis B virus and rhabdovirus^32^,^33^. Specific components in ECM-receptor interaction pathway have been implicated in the host-virus interaction of influenza viruses. A recent study demonstrated the requirement of fibronectin for the entry of influenza A virus^34^. Additionally, a focal adhesion kinase links actin reorganization and thereby regulates influenza A virus entry and replication^35^. The apoptosis signaling modulated influenza A viruses to enable viral replication has been extensively elucidated^36^. Collectively, cellular activities such as adhesion, dynamic behaviors and apoptosis, which are regulated by ECM-receptor interaction, can affect the entry or replication of influenza viruses and thereby influence the predisposition to human A(H7N9) infection.

The influenza A virus infection starts with the attachment of virion to the receptor, followed by the efficient viral propagation in the host cells^37^. Host genes or biological pathways required for viral entry and viral replication have been identified^14^,^38^,^39^. The essential role of MAPK signaling pathway for the replication of influenza A viruses has been explicitly demonstrated^38^. Influenza virus inoculation in transgenic mice with over-activated MAPK pathway resulted in increased disease symptoms and higher mortality^40^. Additionally, specific inhibitors for MAPK pathway displayed antiviral activity against influenza A virus *in vitro* and *in vivo*^41^. The inbred mice study revealed that host genetic components controlling viral replication dynamics was primarily responsible for the host susceptibility to H5N1 diseases^37^. In this GWAS, the identification of MAPK signaling pathway as the significantly enriched category has advanced our understanding that host factors and biological pathways involved in viral replication represent one of critical determinants for human susceptibility to A(H7N9) infection.

There exist limitations in this GWAS. One of them is the very small sample size. Accordingly, the *P* values of most variants were unable to meet the significance threshold for GWAS. However, considering that only about 400 human A(H7N9) influenza have been reported, 102 cases are actually not a small number. Especially we have 106 local healthy poultry workers who have been heavily exposed to A(H7N9) viruses and serve as better controls than general population in this study. Moreover, we are unable to replicate our findings in a replication cohort, which is required for a standard GWAS. A genetic association at the early stage of 2009 pandemic H1N1 influenza encountered the same dilemma, in which 91 cases and 98 controls were applied to a GWAS. In that study, the cutoff *P* value was set as 1 × 10^−4^ for the identification of susceptible variants^42^. Apparently, we have identified the association variants with more stringent criteria. Especially, some of variants identified in this study have been substantiated with functional validation. Nevertheless, further study with more A(H7N9) patients to replicate the identified variants and enriched biological pathways are warranted.

Most A(H7N9) patients primarily manifested as lower respiratory tract infection. Our findings suggest that in affected individuals, the A(H7N9) infection may be attributed to low levels of LGALS1 in the respiratory tract. Additionally, the perturbed ECM-receptor interaction and MAPK signaling pathway, which are involved in viral entry, viral replication and cellular apoptosis, may increase the susceptibility to A(H7N9) infection.

## Methods

### Characteristics of A(H7N9) patients and controls

This study has been approved by the Institutional Review Board of the University of Hong Kong/Hospital Authority of Hong Kong and the institutional review board of the First Affiliated Hospital, College of Medicine, Zhejiang University. Patients with A(H7N9) infection were diagnosed between March 2013 and March 2014. Human A(H7N9) infection was confirmed by reverse transcription-polymerase chain reaction (RT-PCR) and/or viral culture. Acute respiratory distress syndrome and multi-organ dysfunction syndrome were defined with standard criteria^43^,^44^. The healthy poultry workers were sampled from multiple live poultry markets located in 10 districts of Hangzhou, Zhejiang province. Blood and nasal swab samples were collected from them with the written consent. All experiments were carried out in accordance with relevant guidelines and approved protocols.

### Genotyping, quality control and data analysis

Genotyping was genome-widely performed for >890 k SNPs using HumanOmniZhongHua-8 BeadChip (Illumina) according to manufacturer's specification. All participants in the dataset had genotype missing rates <1%. The allelic association *P* values for SNPs were generated using PLINK v1·07^45^. SNPs with more than 10% missing rate or minor allele frequency less than 5% were removed from the association analysis. A total of 705,459 SNPs remained after quality control.

### Imputation analysis, pathway-based analysis and LD analysis

All the variants in our GWAS dataset were applied to the artificial imputation test using the SnipSnip software with default parameters (fixed SNP window size, 10 SNPs; partner matrix, multiplicative). The standard imputation was performed using program MACH v1·0^46^, with genotype data for 286 East-Asian samples (CHB, 97; CHS, 100; JPT, 89) from 1000 Genomes Project released in June 2011 as reference panel. The R package qqman was utilized to create Quantile-Quantile (Q-Q) plot and Manhattan plot^47^. Haploview software was utilized to analyze and visualize the LD pattern of the interested variants^48^. The in-house generated program indelLDplot^49^ (available at https://sourceforge.net/projects/indelldplot/files) was utilized to search for functional SNPs, including indels, in high LD with user-interested variants through mining the publically available 1000 Genomes and HapMap database. A WEB-based GEne SeT AnaLysis Toolkit (WebGestalt) was used to identify the enriched pathways^24^.

### Cells and quantitative expression assays

Lymphoblast cell lines (LCLs) were purchased from Coriell Institute for Medical Research. The cultured LCLs (1 × 10^6^) were fixed with 4% paraformaldehyde for 10 minutes, followed by permeabilization with 0.1% Triton X-100. Cells were then immune-labelled with Rabbit anti-Galectin 1 (LGALS1, Abcam) and secondary antibody Goat anti-Rabbit Alexa 647. The intracellular expression of LGALS1 was determined with a BD FACSCanto II flow cytometer and data were analyzed with FlowJo (TreeStar). Peripheral blood mononuclear cells preparation, monocyte separation and RT-qPCR assay were performed as described previously^13^.

### eQTL analysis and lung eQTL dataset

The linear regression analysis incorporated in PLINK was used to examine the correlation of a specific *LGALS1* genetic variant and the quantitative expression in LCLs, human cells and tissues, using an addictive model to estimate the effect of one copy increment of the variant. A *P* value ≤ 0.05 was regarded as statistically significant. Lung eQTL dataset was generated as described previously^20^. All patients who donated their lung tissue samples for generation of this dataset have provided written informed consent.

## Supplementary Material

Supplementary Information Supplementary materials

## Figures and Tables

**Table 1:** Demographic and clinical characteristics of A(H7N9) patients

Characteristics^a^	Death(n = 27)	Survival(n = 75)	*P*-value^d^
**Demographics**			
Age (years)	66 (61–72)	58 (46–66)	0.002
Female sex	8 (29.6)	27 (36.0)	0.640
**Risk conditions**^b^			
Age ≥ 65	15 (57.7)	24 (32.0)	0.034
Pregnant women	0 (0)	1 (1.3)	1.000
Chronic pulmonary diseases	4 (14.8)	2 (2.7)	0.041
Chronic cardiac diseases	3 (11.1)	4 (5.3)	0.378
Metabolic disorders	4 (14.8)	5 (6.7)	0.240
Chronic renal diseases	1 (3.7)	3 (4.0)	1.000
Chronic hepatic diseases	0 (0)	1 (1.3)	1.000
Neurological conditions	2 (7.4)	4 (5.3)	0.654
Immunosuppression	3 (11.1)	2 (2.7)	0.114
**Laboratory findings on admission**			
Hemoglobin (g/L)	108 (90–125)	124 (110–135)	0.014
Total white blood cell (×10^9^ cells/L)	5.4 (1.8–11.4)	4.1 (3.0–6.1)	0.428
Neutrophil (×10^9^ cells/L)	3.4 (1.4–10.2)	3.3 (2.1–4.9)	0.776
Lymphocyte (×10^9^ cells/L)	0.60 (0.30–0.80)	0.50 (0.40–0.70)	0.272
Platelet (×10^9^ cells/L)	98 (61–184)	128 (93–162)	0.262
Prothrombin time (s)	13.0 (12.1–14.3)	12.5 (12.0–13.6)	0.154
Activated partial thromboplastin time (s)	38.3 (33.7–44.4)	36.0 (30.5–42.2)	0.607
D-dimer (μg/L)	7594 (5780–13900)	2190 (1260–5810)	<0.001
Urea (mmol/L)	8.9 (5.9–16.2)	4.6 (3.3–7.3)	0.001
Creatinine (μmol/L)	88 (70–170)	61 (47–82)	<0.001
Bilirubin (μmol/L)	11.0 (8.8–16.6)	8.0 (6.0–12.0)	<0.001
Alanine transaminase (U/L)	39 (27–65)	33 (21–55)	0.174
Aspartate transaminase (U/L)	87 (39–147)	49 (35–74)	0.002
Lactate dehydrogenase (U/L)	670 (569–873)	394 (309–549)	<0.001
Creatine kinase (U/L)	263 (140–630)	152 (74–301)	0.032
C-reactive protein (mg/L)	113 (78–153)	57 (28–102)	0.001
**Clinical Outcome**			
ICU admission	26 (96.3)	68 (90.7)	0.678
APACHE II score^c^	27 (25–30)	18 (16–22)	<0.001
ARDS	25 (92.6)	49 (65.3)	0.006
MODS	23 (85.2)	10 (13.3)	<0.001
ECMO	6 (22.2)	8 (10.7)	0.190

APACHE II, Acute Physiology and Chronic Health Evaluation II; ARDS, acute respiratory distress syndrome; ECMO, extracorporeal membrane oxygenation; ICU, intensive care unit; MODS, multi-organ dysfunction syndrome.

^a^All continuous variables are expressed as median (interquartile range).

^b^Obesity or hemoglobinopathy were not present in any individuals.

^c^Data only included patients who were admitted to the intensive care unit.

^d^Fisher exact test and Mann Whitney U test were used for categorical variables and continuous variables, respectively.

**Table 2:** The top association anchor-partner SNPs identified with SnipSnip implementation

Anchor-partner SNP	Anchor-partner Position (hg19)	Annotation	Artificial imputation *P*
rs646606|rs706481	57416633|57415200	C8B;chromosome 1 (intronic/intronic)	6.72 × 10^−8^
rs13057866|rs9622682	38069622|38074434	LGALS1; chromosome 22 (upstream/intronic)	1.81 × 10^−7^
rs4820294|rs2899292	38071043|38077718	LGALS1; chromosome 22 (upstream/intergenic)	2.13 × 10^−7^
rs62236673|rs2899292	38076063|38077718	LGALS1; chromosome 22 (downstream/intergenic)	4.25 × 10^−7^

**Table 3:** The haplotype analysis of rs4820294/rs2899292 for disease association in GWAS and *LGALS1* expression association in lymphoblast cell lines (LCLs)

	Disease Association				Expression Association in LCLs	
	Freq					
Haplotype	Case	Control	OR (95% CI)	*P*	Freq	*P*
AA	0.0207	0.0115	2.30 (0.34–15.50)	0.452	0.0277	6.86 × 10^−3^
GA	0.4793	0.4225	1.28 (0.86–1.91)	0.245	0.5871	0.126
AG	0.3567	0.2102	2.10 (1.34–3.31)	9.03 × 10^−4^	0.1817	0.465
GG	0.1433	0.3558	0.26 (0.15–0.45)	5.92 × 10^−7^	0.2034	0.050

Freq, frequency; OR (95% CI), odds ratio (95% confidential interval).

**Table 4:** Enriched biological pathways in A(H7N9) patients versus healthy poultry workers with gene set analysis

Pathway name	ECM-receptor interaction	MAPK signaling pathway
	laminin, alpha 4 (LAMA4)	fibroblast growth factor 1 (FGF1)
	collagen, type IV, alpha 1 (COL4A1)	calcium channel, voltage-dependent, R type, alpha 1E subunit (CACNA1E)
	synaptic vesicle glycoprotein 2B(SV2B)	Rap guanine nucleotide exchange factor 2 (RAPGEF2)
	thrombospondin 3 (THBS3)	fibroblast growth factor 20 (FGF20)
	integrin, beta 4 (ITGB4)	phospholipase A2, group V (PLA2G5)
	CD36 (thrombospondin receptor)	fibroblast growth factor 12 (FGF12)
	integrin, alpha 4(ITGA4)	mitogen-activated protein kinase 8 interacting protein 3 (MAPK8IP3)
	CD47	transforming growth factor, beta 2 (TGFB2)
		mitogen-activated protein kinase kinase kinase 7 (MAP3K7)
		neurotrophic tyrosine kinase, receptor, type 2 (NTRK2)
		calcium channel, voltage-dependent, alpha 2/delta subunit 1 (CACNA2D1)
		RAS p21 protein activator 1 (RASA1)
Ratio of Enrichment	7.76	3.69
Adjusted *P*	0.0008	0.0045

Ratio of enrichment represents the ratio of the number of genes in the gene set and also in the category versus the expected number of genes by chance. The adjusted *P* is the *P* value after correction for multiple tests.
