# Supplementary material for: Functional variants regulating LGALS1 (Galectin 1) expression affect human susceptibility to influenza A(H7N9)
Source: Sci Rep. 2015 Feb 17;5:8517. doi: 10.1038/srep08517 (PMC4649671; doi:10.1038/srep08517)
Supplement: Supplementary Information — Supplementary materials [file srep08517-s1.doc]

Title: Functional variants regulating *LGALS1 (Galectin 1)* expression affect human susceptibility to influenza A(H7N9)

Yu Chen^1,2*^, Jie Zhou^3,4,5*^, Zhongshan Cheng^4^, Shigui Yang^1,2^, Hin Chu^3,4^, Yanhui Fan^12^, Cun Li^4^, Bosco Ho-Yin Wong^4^, Shufa Zheng^1,2^, Yixin Zhu^1,2^, Fei Yu, Yiyin Wang^1,2^, Xiaoli Liu^1,2^, Hainv Gao^1,2^, Liang Yu^1,2^, Linglin Tang^1,2^, Dawei Cui^1,2^, Ke Hao^8^, Yohan Bossé^9^, Ma’en Obeidat^10^, Corry-Anke Brandsma^11^, You-Qiang Song^7^, Kelvin Kai-Wang To^3,4,5,6^, Pak Chung Sham^12^, Kwok-Yung Yuen^3,4,5,6#^, Lanjuan Li^1,2#^

**Supplementary materials.**


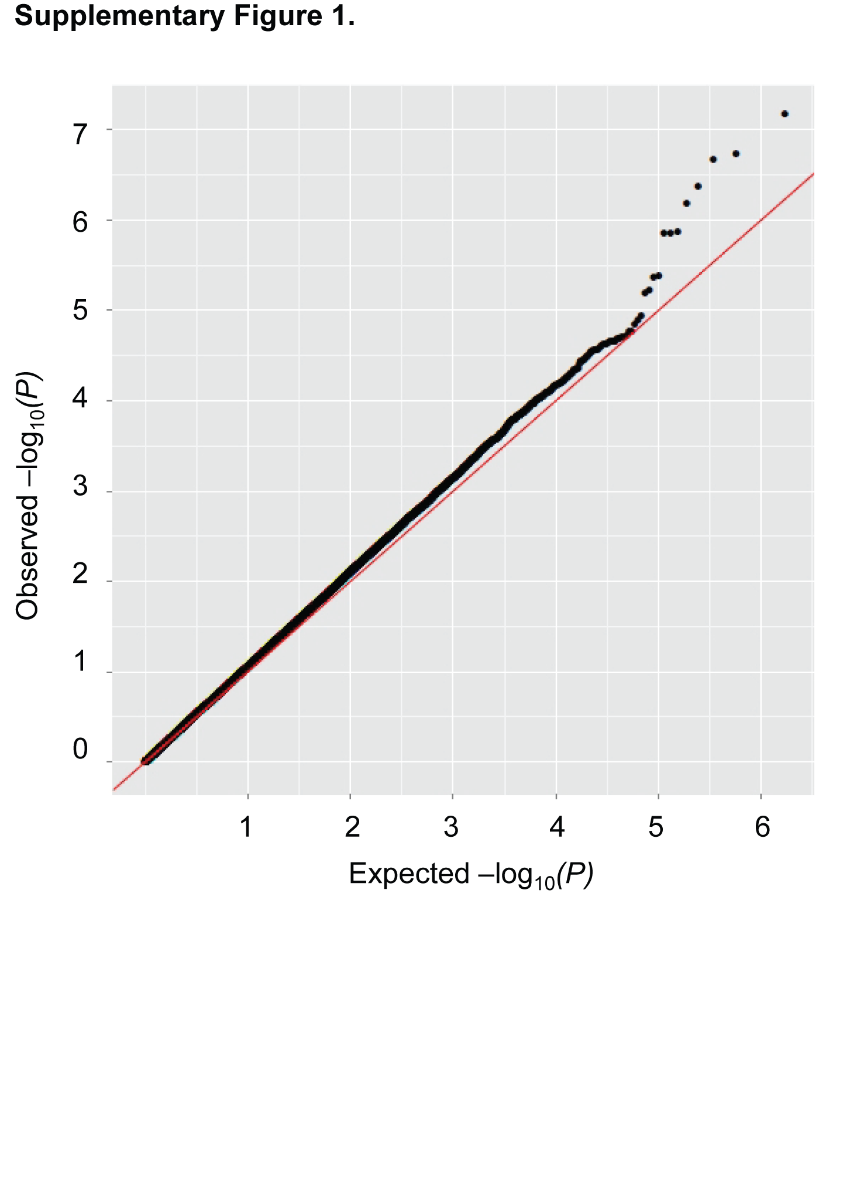


Supplementary Figure 1. The Quantile-Quantile (Q-Q) plot of the variants generated from SnipSnip implementation. The observed –log_10_ *P* values are ranked from smallest to largest on the y-axis and plotted against the distribution that would be expected under the null hypothesis of no association on the x-axis.

Supplementary Table 1. The SNP numbers and the allelic association *P* values of top variants of *LGALS1* and *C8B* after standard imputation.

| *LGALS1* | |  | *C8B* | |
| --- | --- | --- | --- | --- |
| rs71646553 | 2.72E-06 |  | rs1960384 | 2.07E-06 |
| rs9619699 | 2.72E-06 |  | rs7553563 | 2.07E-06 |
| rs58519213 | 2.72E-06 |  | rs72670376 | 2.88E-06 |
| rs34195652 | 2.75E-06 |  | rs139781264 | 2.88E-06 |
| rs2071769 | 2.75E-06 |  | rs72670362 | 3.62E-06 |
| rs13057866 | 2.75E-06 |  | rs72670363 | 3.62E-06 |
| rs34680058 | 5.06E-06 |  | rs56023405 | 4.40E-06 |
| rs71324870 | 1.26E-05 |  | rs72670373 | 4.40E-06 |
| rs78684352 | 1.34E-05 |  | rs199953465 | 4.77E-06 |
| rs9622672 | 7.45E-05 |  | rs515493 | 8.01E-06 |
| rs6000853 | 9.13E-05 |  | rs661586 | 8.01E-06 |
| rs2051593 | 9.13E-05 |  | rs689342 | 9.50E-06 |
| rs13053394 | 9.13E-05 |  | rs630417 | 1.34E-05 |

Supplementary Table 2. Functional annotation of rs13057866, rs4820294 and the variants in high linkage disequilibrium.
